# Nodal melanosis in malignant melanoma: a rare case from the Middle East with comprehensive surgical and histopathological findings

**DOI:** 10.1093/jscr/rjae591

**Published:** 2024-09-18

**Authors:** Alsadig Suliman, Reem Mohamed Osman, Lina SeedAhmed

**Affiliations:** Department of General Surgery, Sudan Medical Specialization Board, Isbitalia Street, Downtown, Khartoum, Khartoum 13315, Sudan; Department of General Surgery, Al-Neelain University, Zubeir Pasha Street, Downtown, Khartoum, Khartoum 11115, Sudan; General Surgery, Wad Madani Teaching Hospital, Hospital Street, Downtown, Wad Madani 21111, Sudan

**Keywords:** malignant melanoma, nodal melanosis, below-knee amputation, inguinal lymph node dissection, distant metastases

## Abstract

Malignant melanoma, a highly aggressive skin cancer, accounts for 75% of skin cancer-related deaths. This case report details a 59-year-old Sudanese male with a malignant melanoma of the left foot, presenting with nodal melanosis, a rare condition involving benign pigmented deposits in the lymph nodes. The patient underwent below-knee amputation and inguinal lymph node dissection. Significant black discoloration was observed in the lymph nodes, indicating nodal melanosis. Histopathological examination of excised lymph nodes confirmed the presence of both malignant melanoma and benign melanosis. Despite surgical intervention, the patient eventually developed distant metastases, including lung nodules and a liver lesion. This case underscores the importance of recognizing nodal melanosis in metastatic malignant melanoma and highlights the challenges of managing advanced cases in resource-limited settings. Surgical management remains critical, particularly wide resection and lymph node dissection, even as systemic therapies advance.

## Introduction

Malignant melanoma (MM) of the skin is the most lethal form of skin cancer, with a 94% 5-year survival rate if localized and a 30% 5-year survival rate in metastatic cases [1]. Globally, MM accounts for 3.2% of all cancers, with varying incidences in different regions; it is more common in developed countries compared to Africa [2–4]. MM is uncommon in the African population and often presents in advanced stages with metastasis [5]. Regional lymph node (LN) metastasis develops in 15–20% of MM, and it is considered the most important prognostic factor, as it’s usually associated with poor prognosis [6]. It commonly affects the lower limbs, especially in females [3]. When MM is located in the distal leg and foot, it typically metastasizes to the inguinal lymph nodes (ILNs) [6].

Tumoral or Nodular melanosis is a benign condition describing a pigmented lesion in the skin that resembles MM, and when it involves the LNs, it’s called nodal melanosis (NM) [7]. It’s a rare variant of completely regressed melanoma, but it can rarely present together with metastatic MM [8]. Histopathology of NM shows the presence of melanophages which stain positively for CD68, but negative for melanocytic markers (S100, HMB-45, Melan-A) [9]. Less than 10 cases were reported in literature discussing NM, with the last one reported in 2017 discussing NM associated with MM [8]. This is the first case to be reported from the Middle East of complicated MM of the left foot presenting with metastases to the left ILNs with NM, which were subsequently excised [7–12].

## Case report

A 59-year-old Sudanese male was admitted to the hospital with a progressive and insidious onset of swelling on the plantar aspect of his left foot, causing difficulty in walking. He also reported lymphadenopathy in his left groin and weight loss. On examination, the swelling was firm, brownish, 6 cm × 7 cm, with plantar ulceration. His vital signs and laboratory results were within normal limits.

A general examination was normal; however, screening revealed enlarged ulcerated left ILNs. Left foot biopsy confirmed nests of abnormal cells in the epidermis and subcutaneous tissue, with pigmentation consistent with MM. Preoperative screening showed no evidence of distant metastasis. Based on these findings, a preoperative diagnosis of left leg MM with regional metastasis was established. Due to extensive tumor spread, and loss of ankle function, a multidisciplinary team recommended below-knee amputation (BKA) with inguinal lymph node dissection (ILND), after careful counseling and obtaining patient consent.

**Figure 1:**
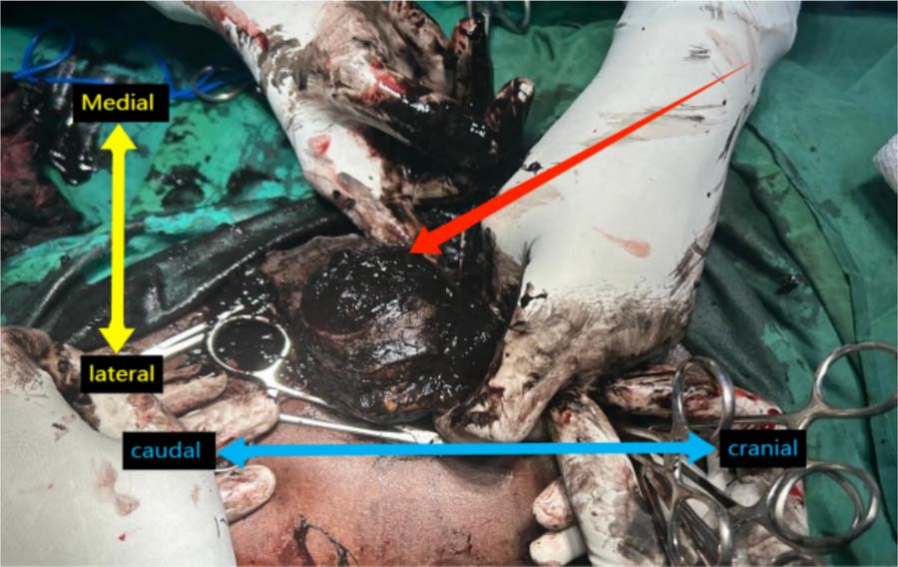
Intra-operative view of the left inguinal region with dark black, pigmented LNs. Orientation with the terms “Medial,” ”Lateral,” “Cranial,” and “Caudal” labeled.

The surgery included a BKA and ILND. The ILND was performed through an elliptical incision, during which the surgical field was notably dark black, with all LNs significantly enlarged, and prominent black discoloration was observed an extremely unusual finding (Fig. 1).

**Figure 2:**
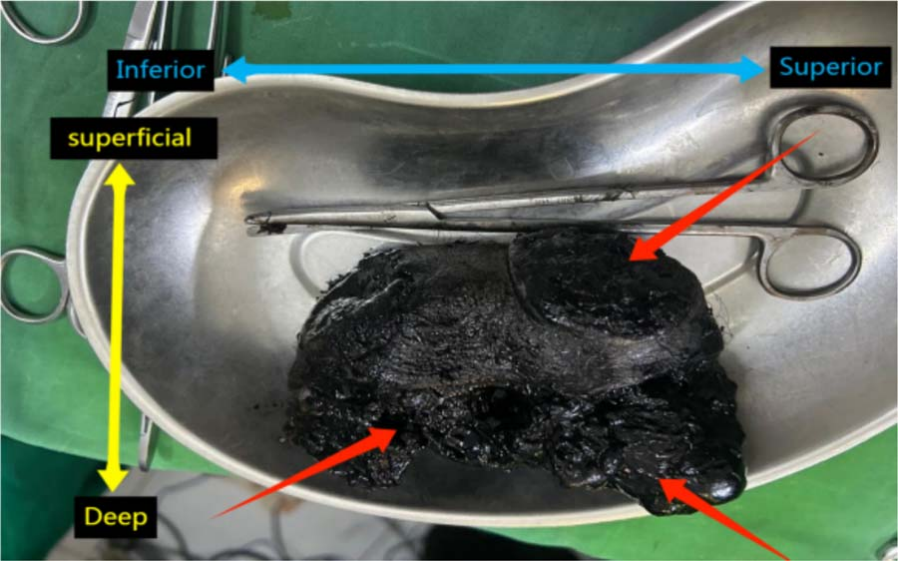
Specimen of ILND showing an enlarged black pigmented LN. Anatomical orientation is indicated with “Superior,” “Inferior,” “Superficial,” and “Deep” labeled.

Macroscopic examination revealed multiple enlarged LNs, with the largest measuring 3.5 cm in diameter. The nodes displayed a dark black, pigmented, firm surface (Fig. 2). Histological examination of the ILNs revealed typical cancerous features consistent with MM, alongside heavily pigmented macrophages which were identified as NM. Overall, the diagnosis is metastatic MM accompanied by NM.

Following the histological findings, the patient was referred to an oncology center for further chemoradiotherapy. However, the patient discontinued follow-up and re-presented ~6 months post-surgery with lung and liver metastases before eventually losing follow-up.

## Discussion

MM originating from melanocytes is one of the most aggressive forms of cancer. While relatively rare compared to other skin cancers, MM accounts for ~75% of all skin cancer deaths due to its aggressive nature and tendency to spread [8].

NM is a rare condition observed in LNs, characterized by the presence of melanophages without evidence of active MM [10]. The accumulation of melanophages in the LNs may indicate metastasis of malignant melanocytic cells that have been phagocytosed by macrophages or could reflect the migration of melanophages from the primary lesion to the affected LNs [8]. To our knowledge, only six cases of NM have been described, with just three involving primary cutaneous MM [8, 10, 11]. In two of them, the primary MM was fully regressed and only one case reported MM containing malignant cells with no regression. In our case, the primary lesion also contained malignant melanocytes positive for melanocytic markers with no regression. Regarding the prognosis of NM, it’s not well defined, due to the limited number of cases. However, Solano-Lopez et al. suggested that NM should be considered a poor prognostic factor and should be managed as a high-risk MM [11]. This was attributed to the fact that the patient after lymphadenectomy developed distant metastasis in the following years and died [11]. This comes in agreement with our case, in which the patient developed distant metastasis to the lung and liver after a few months.

Immediate LN dissection has been shown to improve 5-year survival rates by 20% in patients with metastatic MM compared to those who undergo delayed lymphadenectomy [12, 13].

While ILND can reduce local recurrence, it has not shown a definitive survival benefit and is associated with significant postoperative morbidity, such as wound infections, seroma formation, and skin necrosis [14]. The surgical approach to ILND includes superficial and deep dissections, typically performed through elliptical, transverse, vertical, oblique, or Z-shaped incisions, depending on the extent of dissection and anatomical considerations [14]. Despite advances in systemic treatments, surgical resection remains the cornerstone of managing primary and metastatic MM, with recent innovations focusing on minimizing surgical morbidity and improving patient outcomes [15].

## Conclusion

Our case report emphasizes the need to consider MM in the differential diagnosis, even in populations where it is less common. NM is a poor prognostic factor and should be treated as a high-risk form of MM. The aggressive nature of MM, as demonstrated by this patient's treatment with BKA and ILND, highlights the necessity of comprehensive management. Immediate lymphadenectomy is especially crucial in low-resource settings to improve patient outcomes. Further call for more research into the pathogenesis and management of NM is needed, with a focus on education and early diagnosis.
